# Pulmonary Tuberculosis-Associated Morbidity in Africa: A Systematic Review

**DOI:** 10.7759/cureus.77569

**Published:** 2025-01-17

**Authors:** Collins C Okeke, Chiemezie C Ibe, Oluchi J Nduji, Chizoba J Ndulue, Onyinye Ngige, Chukwunomso C Amuchie, Chinecherem C Ezema, Afamefuna O Onyeogulu, Angela Ojo, Michael Obuseh, Omosimisola O Alli, Emmanuel I Akinteye, Kris N Idion, Ubon I Akpan-Udo, Isabella C Okereke, Chibueze Eke

**Affiliations:** 1 Internal Medicine, University of Port Harcourt Teaching Hospital, Port Harcourt, NGA; 2 Oncology, North Middlesex University Hospital, London, GBR; 3 Paediatrics, Abia State University, Okigwe, NGA; 4 General Practice, Chukwuemeka Odumegwu Ojukwu University College of Medicine, Awka, NGA; 5 Internal Medicine, Nnamdi Azikiwe University Teaching Hospital, Nnewi, NGA; 6 General Practice, Accra College of Medicine, Accra, GHA; 7 Integrative Medicine, Nnamdi Azikiwe University Teaching Hospital, Anambra, NGA; 8 Internal Medicine, Afe Babalola University, Ado-Ekiti, Ado Ekiti, NGA; 9 Internal Medicine, Delta State University Teaching Hospital, Delta, NGA; 10 General Practice, Lagos State University, College of Medicine, Lagos, NGA; 11 Internal Medicine, Bowen University Teaching Hospital, Iwo, NGA; 12 Internal Medicine, Asaba Specialist Hospital, Asaba, Asaba, NGA; 13 Family Medicine, University of Uyo Teaching Hospital, Uyo, NGA; 14 Internal Medicine, Igbinedion University Okada, Lagos, NGA; 15 Internal Medicine, Federal University Teaching Hospital Owerri, Owerri, NGA

**Keywords:** africa, complication, morbidity, pulmonary tuberculosis, sequelae

## Abstract

Tuberculosis (TB) remains a global health problem despite the availability of effective medications and preventive measures. TB in humans is caused by *Mycobacterium tuberculosis* (MTB) and usually affects the lungs causing pulmonary TB. It is transmitted via *M. tuberculosis*-containing aerosol droplets and nuclei. TB has a devastating effect on Africa and this review aims to identify the most common morbidities associated with and the sequelae/complications of pulmonary TB in Africa and also explore a management option to decrease the impact of morbidity/complications and increase quality of life.

A comprehensive search conducted from inception to the 5th of December 2024 on PubMed, Scopus, and African Journal Online returned 6312 for which 1441 duplicates were removed. Four thousand, eight hundred seventy-one articles were screened and 4791 articles were removed following title and abstract screening, 80 articles were subjected to full-text screening, and ultimately, 17 articles met the requirements for the inclusion criteria which included original articles published in English in peer-reviewed journals from inception to November 2024 that reported morbidity/sequelae among patients of all ages and genders in Africa diagnosed with pulmonary TB via culture and currently receiving treatment and or have completed standardized treatment.

From the 17 articles, a total of 4552 patients were included from 11 different African countries. There were 2358 males and 1918 females diagnosed with pulmonary TB for which mental illness was the most reported morbidity (depression, psychological distress, generalized anxiety, adjustment disorder, schizophrenia, psychosis) followed by lung impairment and then airway obstruction. Other comorbidities were HIV, asthma, and chronic obstructive pulmonary airway disease, diabetes, chicken pox, and heart-related disease.

This study highlighted the profound impact of TB on mental health, respiratory function, and systemic health exacerbated by comorbidities such as HIV, diabetes, and chronic pulmonary conditions. Additionally, the review reveals unique morbidities like secondary amenorrhea and medication-induced psychosis, often overlooked in global TB research.

## Introduction and background

Despite the availability of effective medications and preventive measures, tuberculosis (TB) remains a deeply entrenched global health problem. Gram-positive, acid-fast bacilli belonging to the genus Mycobacterium are the cause of TB, a progressive granulomatous infectious illness. *Mycobacterium tuberculosis* is the main cause of TB in humans, which mostly affects the lungs and results in pulmonary TB. It can also cause extra-pulmonary TB by affecting the skin, lymph nodes, bones, joints, gut, meninges, and other bodily parts. The primary methods of human TB transmission are droplet infection and droplet nuclei [1]. Globally, individuals are afflicted by the deadly infectious TB. Even though low- and middle-income nations account for almost 80% of occurrences, TB is nevertheless a problem globally.

In 2022, 46% of new cases of TB were in the Southeast Asian Region, according to the World Health Organization (WHO). Africa came in second at 23%, while the Western Pacific area had 18% of new cases [2].

An estimated 25% of people worldwide are thought to have contracted TB. Of those infected with TB, 5-10% will eventually develop symptoms and TB illness. The number of deaths from TB in 2023 was 1.25 million, including 161,000 HIV-positive individuals. In addition to being the largest cause of death for individuals with HIV and a significant contributor to antimicrobial resistance, TB has likely reclaimed its position as the world’s leading infectious agent-related cause of death. An estimated 10.8 million persons worldwide contracted TB in 2023, comprising 1.3 million children, 3.6 million women, and 6.0 million men. All nations and age groups are affected by TB. TB is preventable and cured. According to the 2023 UN high-level level meeting on TB, approximately 22 billion US dollars are required each year for TB prevention, diagnosis, treatment, and care in order to fulfill the worldwide objective by 2027 [3].

TB is the most common infectious agent and the ninth-largest cause of death globally, surpassing HIV/AIDS. Africa accounts for more than one-third of TB-related deaths. Multidrug-resistant TB (MDR-TB) is a danger to health security and raises the possibility of undoing progress in the fight against TB [2]. There were an expected 424,000 TB deaths (1.267 million worldwide) and 2.5 million TB illnesses in the African continent in 2022, which accounted for a quarter of all new TB cases worldwide. In Africa, TB accounts for more than one-third of all fatalities [2]. Between 2015 and 2022, the incidence of TB decreased by 23% globally and by -8.7% in the African region. This must be accelerated in order to meet the End TB Strategy’s 2025 goals, which include a 50% decrease in incidence from 2015. Between 2010 and 2022, TB diagnosis and treatment are predicted to have saved 44 million lives worldwide, with 10 million of those lives occurring in the African region [2]. Nigeria still holds the position of the country with the highest TB burden in West Africa with an absolute estimated incidence of 407,000 cases in 2016 [4]. Tanzania, Kenya, and Uganda are among the high TB burden countries in East Africa with reported TB incidence rates of 292, 253, and 200 per 100,000 population respectively in 2018 [5]. In North Africa, the highest prevalence rates of TB were in Egypt at 28935.42 [6]. South Africa has a particularly high burden of TB, with an incidence rate of 468 per 100,000 of the population [7].

Active TB patients can spread the disease by coughing, sneezing, or talking, which releases aerosol droplets and nuclei containing MTB. The TB bacteria enter the new host's lungs through the respiratory system after being inhaled. Alveolar macrophages then internalize the tubercle bacilli while the host's innate immune system comes in to ward off the infection. When the macrophages fail to inhibit or destroy the bacilli, the bacteria multiply within their intracellular environment, get released, and then are phagocytosed again by other alveolar macrophages and the cycle continues. Following the recruitment of lymphocytes to the infection site, a swarm of immune cells comes in an effort to contain the bacteria and prevent further growth, initiating a cell-mediated immune response. The host is still asymptomatic at this point, and the TB bacteria may either go into latency inside the granuloma or be totally eradicated. However, in the setting of impaired immunity, the disease immediately progresses into active TB with clinical symptoms [8]. Common symptoms of active lung TB are cough with sputum and blood at times, chest pains, weakness, weight loss, fever, and night sweats [9]. WHO recommends the use of rapid molecular diagnostic tests as the initial diagnostic test in all persons with signs and symptoms of TB while tuberculin skin test (TST), interferon-gamma release assay (IGRA), or newer antigen-based skin tests (TBST) can be used to identify people with infection. TB is treated with special antibiotics, commonly used are: isoniazid, rifampicin, pyrazinamide, and ethambutol. For effectiveness, medications need to be taken daily for 4-6 months [3].

This systematic review aims to identify the most common morbidities associated with and the sequelae/complications of pulmonary TB in Africa and also explore a management option to decrease the impact of morbidity/complications and increase quality of life.

## Review

Methods

The Preferred Reporting Items for Systematic Reviews and Meta-Analysis (PRISMA) extension for systematic reviews [10] were followed in the course of this systematic review. The study protocol was registered with Prospero CRD42024625260.

Inclusion Criteria

We included original articles published in English in peer-reviewed journals from inception to November 2024 that reported morbidity/sequelae among patients of all ages and genders in Africa diagnosed with pulmonary TB via culture and currently receiving treatment and or have completed standardized treatment.

Exclusion Criteria

Patients were managed in a hospital outside Africa, and study designs such as case reports, audits, opinions, reviews, meta-analyses, letters, comments, and editorials were excluded.

A comprehensive search was conducted from inception to the 5th of December 2024, on PubMed, Scopus, and African Journal Online(AJOL). The keywords used were ((Pulmonary tuberculosis) AND (Morbidity)) AND (Africa), ((Pulmonary tuberculosis) AND (complications)) AND (Africa), "Pulmonary Tuberculosis" "Morbidity" "Africa" and "Pulmonary Tuberculosis" "Complication" "Africa" as shown in the Appendix Table 3.

Three independent reviewers (C.C.O, C.C.I, O.J.N) performed duplicate, title, and abstract screening against the predefined eligibility criteria using the Rayyan systematic review software [11]. Potentially eligible studies were screened for full-text review. Disagreements were discussed among reviewers; in the case of no resolution, an appeal was made to another reviewer (C.J.N).

We extracted data from articles related to the author, study year, study design, sample size, mean age, gender, anti-TB medication, comorbidity, and morbidity. The risk of bias in included studies was assessed using the JBI critical appraisal tool for cross-sectional and cohort. JBI’s critical appraisal tools assist in assessing published papers’ trustworthiness, relevance, and results. This appraisal aims to assess a study's methodological quality and determine the extent to which a study has addressed the possibility of bias in its design, conduct, and analysis. Articles are assessed with a yes, no, unclear, and not applicable as shown in the appendix Tables 4-5 [12]. The randomized control trials were accessed using the Scottish Intercollegiate Guidelines Network (SIGN) assessment tool [13] and articles were assessed with a yes, no, and can’t say. A ++ is assigned to a high-quality article, + is assigned to an acceptable article, a - is assigned to a poor-quality article and 0 is assigned to an unacceptable article, as shown in Appendix Table 6.

Results

**Figure 1:**
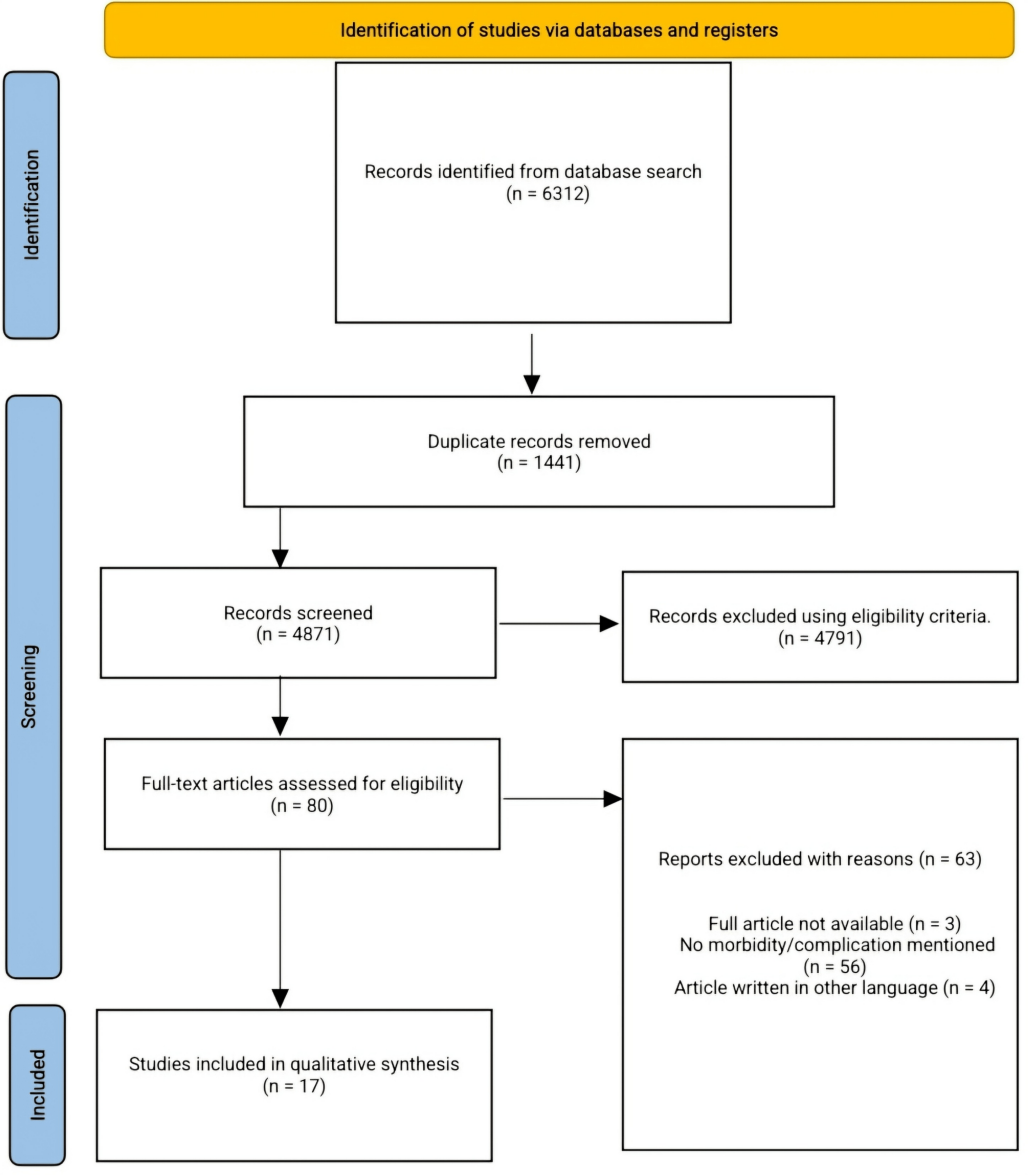
PRISMA flow diagram The image was created by the authors of this article.

Our search returned 6312 articles, 1441 articles were removed following duplicate screening and 4871 were screened. 4791 articles were excluded after the title and abstract screening using the eligibility criteria, and 80 articles were subjected to full-text screening to determine their eligibility based on our inclusion criteria. Ultimately, 17 articles were included in the final qualitative synthesis. Exclusions were made due to various reasons, including failure to meet our inclusion criteria, unavailability of full articles, unavailability of morbidity/complications of pulmonary TB mentioned in an article, and articles written in other languages (French language). Figure 1 displays the PRISMA flow diagram.

Study characteristics

A total of 4552 patients were included in 17 articles, from 11 countries in Africa (Cameroon, South Africa, Ethiopia, Zambia, Nigeria, Gambia, Uganda, Egypt, Lesotho, Zimbabwe, and Tanzania). South Africa produced the highest number of articles (5), followed by Nigeria (3), then Ethiopia, Uganda, and Zambia producing two articles each. The study period ranged from 1996 to 2021 and the mean age of patients reported ranged from 1.1 to 50 years across the studies. There were 2358 males and 1918 females diagnosed with pulmonary TB. The study characteristics of the included articles are shown in Table 1.

**Table 1:** Study characteristics

Author	Year	Country	Sample size	Mean age	Male	Female
Kehbila et al. [14]	2015	Cameroon	265	37	129	136
Osman et al. [15]	2017	South Africa	51	40	32	19
Ayana et al. [16]	2018	Ethiopia	365	36	205	160
Hestad et al. [17]	2019	Zambia	38	34	25	13
Ilesanmi et al. [18]	2017	Nigeria	152	41	85	67
Philips et al. [19]	2014	South Africa	59	33	48	11
Nkereuwem et al. [20]	2019	Gambia	68	6.5	36	32
Binegdie et al. [21]	2013	Ethiopia	134	40	72	62
Namusobya et al. [22]	2021	Uganda	162	30	97	65
Kirenga et al. [23]	2013	Uganda	365	29	158	207
Aghanwa and Erhabor [24]	1996	Nigeria	53	37	24	29
Mkoko et al. [25]	2016	South Africa	173	50	103	70
Goussard et al. [26]	2011	South Africa	250	1.1	144	106
Lasebikan and Ige [27]	2014	Nigeria	115	35	44	71
Hassan and Darwish [28]	2008	Egypt	429	41	85	67
Hayes-Larson et al. [29]	2015	Lesotho	371	35	212	160
Theron et al. [30]	2012	South Africa, Zimbabwe, Zambia, and Tanzania	1502	37	859	643

The study with the largest sample size was 1502 [30], while 38 was the smallest sample size in these studies [17]. The study types included in this review are cross-sectional studies [14-23], prospective and retrospective cohort studies [24-27], randomized control trials [28], mixed-method cluster-randomized trials [29], and randomized parallel-arm, multi-centric trials [30].

Morbidity

From the included articles, mental illness was the most reported morbidity(depression, psychological distress, generalized anxiety, adjustment disorder, schizophrenia, psychosis), followed by lung impairment, and then airway obstruction. Other reported morbidity can be seen in Table 2. HIV, asthma, and chronic obstructive pulmonary airway disease were the most common comorbidities reported in this study [14-18,20,22,23,25,26,29,30]. Other comorbidities included diabetes [23], pneumonia [26], chicken pox [22], and heart-related disease [14].

**Table 2:** Morbidities of pulmonary tuberculosis

Author	Morbidity
Kehbila et al. [14]	Depression
Osman et al. [15]	Shortness of breath, severe chest illness, wheezing
Ayana et al. [16]	Psychological distress
Hestad et al. [17]	Cognitive impairment
Ilesanmi et al. [18]	Depression
Philips et al. [19]	Insulin resistance
Nkereuwem et al. [20]	Lung impairment
Binegdie et al. [21]	Post-TB fibrosis, post-TB bronchiectasis, aspergilloma, post-TB granuloma pleural thickening, tuberculosis-related lung destruction
Namusobya et al. [22]	Chronic pulmonary aspergillosis
Kirenga et al. [23]	Parenchymal nodules, infiltrates, masses p parenchymal cavities pleural effusion
Aghanwa and Erhabor [24]	Depression, generalized anxiety, adjustment disorder
Mkoko et al. [25]	Chronic lung disease
Goussard et al. [26]	Airway obstruction
Lasebikan and Ige [27]	Schizophrenia, non-affective psychosis, medication-induced psychotic disorder
Hassan and Darwish [28]	Secondary amenorrhea, hypomenorrhea, intermenstrual spotting, menorrhagia, dysmenorrhea
Hayes-Larson et al. [29]	Depression, harmful alcohol use
Theron et al. [30]	Psychological distress

Discussion

This systematic review synthesizes data from 17 studies across 11 African countries, encompassing 4552 patients, and provides an in-depth analysis of the morbidities and comorbidities associated with pulmonary TB. The findings align with existing global evidence and reveal unique regional and demographic patterns, offering valuable insights for clinical practice and public health interventions in African contexts. Pulmonary TB presents with varying morbidity as seen in this study which includes psychological illness(depression, psychological distress, schizophrenia, non-affective psychosis, medication-induced psychotic disorder, generalized anxiety, adjustment disorder), respiratory/lung impairments (lung impairment, post-TB fibrosis, post-TB bronchiectasis, aspergilloma, post-TB granuloma, pleural thickening, TB-related lung destruction, parenchymal nodules, parenchymal infiltrates, parenchymal masses, parenchymal cavities, pleural effusion, airway obstruction, chronic lung disease, shortness of breath and wheezing), also rare morbidities like cognitive impairment, insulin resistance, harmful alcohol use then menstrual abnormalities and comorbidity (HIV, asthma, chronic obstructive pulmonary disease (COPD), pneumonia, diabetes, chicken pox, and heart-related disease) profiles across different economic settings.

Demographic Insights: Gender, Age, and Geography

This study offers valuable insights into the demographic distribution. Male predominance (2358 males vs. 1918 females) aligns with global trends suggesting higher TB incidence in males due to biological and behavioral factors. However, the inclusion of pediatric data, such as the study by Goussard et al. [26] with a mean age of 1.1 years, highlights the often-overlooked burden of TB in children. This contrasts with many studies that predominantly focus on adult populations [31,32]. In high-income countries (HICs), TB disproportionately affects marginalized populations, including immigrants, refugees, and people experiencing homelessness, regardless of gender. Pediatric TB is rare due to effective vaccination programs and low community transmission rates. This contrasts with the findings in low- and middle-income countries (LMICs), which have a greater demographic impact according to the WHO report [2].

Geographically, South Africa’s contribution to five studies reflects its high TB burden and research capacity. The inclusion of data from less-represented countries like Gambia and Lesotho adds diversity to the analysis, addressing gaps in TB research from low-resource settings.

Psychological Morbidities: A Growing Concern

The prominence of mental health disorders, particularly depression, anxiety, and psychological distress, among TB patients in this study, is consistent with global trends. Studies such as those by Pachi et al. [33], have documented the interplay between TB and mental health, emphasizing how stigma, chronic disease burden, and socio-economic factors heighten psychological distress. However, our study expands the scope by including less commonly reported conditions like schizophrenia, psychosis, and medication-induced psychotic disorders, which have been underexplored in the literature. In LMICs, mental health morbidities are often driven by poverty, stigma, and limited access to mental health services. Less commonly reported disorders, including schizophrenia and psychosis, were also highlighted, possibly reflecting the compounded mental health burden in resource-limited settings. Similar trends of depression and anxiety have been documented among TB patients in HICs [33]. However, the severity of mental health outcomes is often lower due to robust mental health infrastructure and earlier detection and intervention. Additionally, the stigma associated with TB is less pronounced in HICs, where TB incidence is lower, and public awareness is higher [33-35]. This highlights the need for targeted mental health screening and interventions in TB care settings, particularly in resource-limited environments where mental health services are often scarce.

Respiratory Morbidities: Granularity in Clinical Outcomes

Respiratory sequelae, including post-TB fibrosis, bronchiectasis, airway obstruction, and chronic pulmonary aspergillosis, emerged as critical long-term complications. These findings are consistent with prior studies such as Pasipanodya et al. [36], which underscore the high prevalence of lung impairment among TB survivors. However, the detailed categorization of respiratory morbidities in this study enhances the clinical understanding of TB's pulmonary impact, particularly in African populations. Although post-TB lung disease is also prevalent in HICs, the severity is typically lower due to earlier diagnosis, more effective treatments, and access to pulmonary rehabilitation programs [36]. Studies in HICs often report milder forms of airway obstruction or restrictive lung disease compared to the advanced structural lung damage seen in LMICs [37,38]. The inclusion of specific conditions like aspergilloma and TB-related lung destruction highlights the need for post-treatment pulmonary rehabilitation programs to mitigate long-term morbidity.

Comorbidities: HIV and Beyond

The high prevalence of HIV co-infection in the reviewed studies corroborates existing epidemiological data from sub-Saharan Africa, where HIV and TB co-epidemics continue to pose significant public health challenges. This is consistent with findings by Suthar et al [31], which demonstrated the synergistic burden of HIV and TB on morbidity and mortality. Beyond HIV, this study identifies a range of comorbidities, including diabetes, pneumonia, and cardiovascular diseases, reflecting the complex interplay of TB with non-communicable diseases. Unlike LMICs, HICs show that diabetes and COPD are the most commonly reported comorbidities, reflecting the aging populations and higher prevalence of non-communicable diseases in HICs [39]. HIV co-infection is less prominent due to widespread access to antiretroviral therapy (ART) and lower HIV prevalence. Other conditions, such as chronic kidney disease and immunosuppression related to organ transplantation, are more relevant in HIC populations [2]. These findings emphasize the importance of integrated care models that address both infectious and chronic conditions to improve patient outcomes.

Recommendation

The findings of this review emphasize the need for a multidimensional approach to addressing pulmonary TB and its associated morbidities, particularly in resource-limited settings. Based on the results, the following recommendations are proposed for improving research, clinical practice, and public health interventions: Integration of mental health services into TB care by incorporating routine mental health screening and support services into TB treatment programs. Mental health interventions, such as cognitive behavioral therapy and pharmacological treatments, can improve adherence to TB medications and patient outcomes. Healthcare workers should be trained to recognize and manage mental health disorders in TB patients, with clear referral pathways to mental health specialists. Strengthening post-treatment pulmonary rehabilitation programs through the development and expansion of post-TB rehabilitation services to address long-term pulmonary complications, such as fibrosis, bronchiectasis, and airway obstruction. Multidisciplinary teams, including pulmonologists and physiotherapists, should be established to provide rehabilitation services, including spirometry monitoring, respiratory exercises, and oxygen therapy.

Enhancing diagnostic and treatment capacity in LMICs by investing in diagnostic infrastructure and access to advanced treatment options to reduce delays in diagnosis and improve outcomes. Scaling up molecular diagnostic tools like GeneXpert MTB/RIF and ensuring equitable access to TB medications.

Expanding research into underexplored TB morbidities by conducting focused research on less commonly reported TB-related morbidities, such as menstrual irregularities, medication-induced psychosis, and secondary infections like chickenpox. Longitudinal cohort studies should track the emergence and progression of these morbidities and investigate their underlying mechanisms.

Prioritizing pediatric TB in research and policy by increasing research and policy focus on pediatric TB, with attention to the unique morbidities and management challenges in children. Developing child-specific TB diagnostic tools, strengthening vaccination programs, and studying the long-term effects of TB on child growth and development are essential steps forward.

Scaling up public health interventions to reduce TB incidence by focusing on preventive strategies, including universal bacillus Calmette-Guérin (BCG) vaccination and production of a new and effective vaccine that will reduce the incidence of TB, improve nutrition, and reduce socio-economic barriers to TB care. Community-based awareness campaigns strengthened TB contact tracing programs, and patient incentives for early diagnosis and treatment adherence are critical components of effective prevention.

Creating integrated care models for chronic TB sequelae via the establishment of integrated care models to address chronic TB-related conditions such as diabetes, COPD, and cardiovascular disease. Coordinated care programs that incorporate TB treatment with chronic disease management should be supported by cross-specialty training for healthcare providers.

Promoting linguistic and geographic inclusivity in research by including studies published in non-English languages to better represent francophone and other underrepresented regions in Africa. Journals should support multilingual submissions and collaborations with researchers from underrepresented linguistic regions through discounts or waivers in research publications.

Study strengths and limitations

The review encompasses a wide range of study designs, including cross-sectional studies, cohort studies, and randomized controlled trials. This methodological diversity strengthens the robustness of the findings, offering both prevalence estimates and insights into causality. However, the exclusion of French-language studies is a limitation that may have introduced regional bias, particularly given the significant TB burden in francophone African countries [40]. Future reviews should strive for linguistic inclusivity to provide a more comprehensive synthesis.

## Conclusions

This study provides a comprehensive synthesis of the morbidities and comorbidities associated with pulmonary TB across African populations, highlighting significant clinical, demographic, and regional insights. The findings underscore the profound impact of TB on mental health, respiratory function, and systemic health, exacerbated by comorbidities such as HIV, diabetes, and chronic pulmonary conditions. Additionally, the review reveals unique morbidities like secondary amenorrhea and medication-induced psychosis, often overlooked in global TB research, and emphasizes the burden of TB among pediatric and underrepresented populations.

The analysis highlights critical disparities in TB outcomes between resource-limited and high-income settings, underscoring the role of healthcare infrastructure, diagnostic capacity, and social determinants in shaping disease outcomes. The persistence of severe respiratory complications, compounded by delays in diagnosis and treatment, calls for a stronger focus on rehabilitation and integrated care models to address chronic sequelae.

## Appendices

**Table 3:** Search strategy AJOL: African Journal Online

Search strategy	Database	Outcome
((Pulmonary tuberculosis) AND (Morbidity)) AND (Africa)	PubMed	3821
((Pulmonary tuberculosis) AND (complications)) AND (Africa)	PubMed	1656
"Pulmonary Tuberculosis" "Morbidity" "Africa"	Scopus	84
"Pulmonary Tuberculosis" "Complication" "Africa"	Scopus	118
"Pulmonary Tuberculosis" "Morbidity" "Africa"	AJOL	511
"Pulmonary Tuberculosis" "Complication" "Africa"	AJOL	122

**Table 4:** JBI assessment tool for cross-sectional studies

Checklist question	Kehbila et al. [14]	Osman et al. [15]	Ayana et al. [16]	Hestad et al. [17]	Ilesanmi et al. [18]	Philips et al. [19]	Nkereuwem et al. [20]	Binegdie et al. [21]	Namusobya et al. [22]	Kirenga et al. [23]
Were the criteria for inclusion in the sample clearly defined?	Y	Y	Y	Y	Y	Y	Y	Y	Y	Y
Were the study subjects and the setting described in detail?	Y	Y	Y	Y	Y	Y	Y	Y	Y	Y
Was the exposure measured in a valid and reliable way?	Y	Y	Y	Y	Y	Y	Y	Y	Y	Y
Were objective, standard criteria used for measurement of the condition?	Y	Y	Y	Y	Y	Y	Y	Y	Y	Y
Were confounding factors identified?	Y	Y	Y	Y	Y	Y	Y	Y	Y	Y
Were strategies to deal with confounding factors stated?	Y	N	N	Y	Y	Y	Y	N	N	N
Were the outcomes measured in a valid and reliable way?	Y	Y	Y	Y	Y	Y	Y	Y	Y	Y
Was appropriate statistical analysis used?	Y	Y	Y	Y	Y	Y	Y	Y	Y	Y

**Table 5:** JBI assessment tool for cohort studies

Checklist question	Aghanwa and Erhabor [24]	Mkoko et al. [25]	Goussard et al. [26]	Lasebikan and Ige [27]
Were the two groups similar and recruited from the same population?	Y	Y	Y	Y
Were the exposures measured similarly to assign people to both exposed and unexposed groups?	Y	Y	Y	Y
Was the exposure measured in a valid and reliable way?	Y	Y	Y	Y
Were confounding factors identified?	Y	Y	Y	Y
Were strategies to deal with confounding factors stated?	Y	Y	Y	Y
Were the groups/participants free of the outcome at the start of the study (or at the moment of exposure)?	Y	Y	Y	Y
Were the outcomes measured in a valid and reliable way?	Y	Y	Y	Y
Was the follow-up time reported and sufficient to be long enough for outcomes to occur?	Y	Y	Y	N
Was the follow-up complete, and if not, were the reasons for the loss of follow-up described and explored?	N	N	Y	N
Were strategies to address incomplete follow-up utilized?	N	N	N	N
Was appropriate statistical analysis used?	Y	Y	Y	Y

**Table 6:** SIGN assessment tool for randomized control trial SIGN: Scottish Intercollegiate Guidelines Network

Checklist question	Hassan and Darwish [28]	Hayes-Larson et al. [29]	Theron et al. [30]
The study addresses an appropriate and clearly focused question.	Y	Y	Y
The assignment of subjects to treatment groups is randomized.	Y	Y	Y
An adequate concealment method is used.	Y	Y	Y
The design keeps subjects and investigators ‘blind’ to treatment allocation.	Y	Y	Y
The treatment and control groups are similar at the start of the trial.	Y	Y	Y
The only difference between groups is the treatment under investigation.	Y	Y	Y
All relevant outcomes are measured in a standard, valid, and reliable way.	Y	Y	Y
What percentage of the individuals or clusters recruited into each treatment arm of the study dropped out before the study was completed? All the subjects are analyzed in the groups to which they were randomly allocated (often referred to as intention to treat analysis).	<20% drop	<20% drop	<20% drop
Where the study is carried out at more than one site, results are comparable for all sites.	Y	Y	Y
How well was the study done to minimize bias?	++	++	++

## References

[1] Khan MK, Islam MN, Ferdous J, Alam MM (2019). An overview on epidemiology of tuberculosis. Mymensingh Med J.

[2] (2024). Tuberculosis (TB) | WHO | Regional Office for Africa [Internet]. https://www.afro.who.int/health-topics/tuberculosis-tb.

[3] (2024). Tuberculosis (TB). https://www.who.int/news-room/fact-sheets/detail/tuberculosis.

[4] Adebisi Y A, Agumage I, Sylvanus T D (2019). Burden of tuberculosis and challenges facing its eradication in West Africa. Int J Infect.

[5] Mnyambwa NP, Philbert D, Kimaro G (2021). Gaps related to screening and diagnosis of tuberculosis in care cascade in selected health facilities in East Africa countries: A retrospective study. J Clin Tuberc Other Mycobact Dis.

[6] Moradinazar M, Afshar ZM, Ramazani U, Shakiba M, Shirvani M, Darvishi S (2023). Epidemiological features of tuberculosis in the Middle East and North Africa from 1990 to 2019: Results from the global burden of disease Study 2019. Afr Health Sci.

[7] (2024). WHO: Intensifying efforts to end TB. https://www.afro.who.int/countries/south-africa/news/south-africa-intensifying-efforts-end-tb.

[8] Alsayed SS, Gunosewoyo H (2023). Tuberculosis: Pathogenesis, current treatment regimens and new drug targets. Int J Mol Sci.

[9] (2024). Tuberculosis [Internet]. Africa CDC. https://africacdc.org/disease/tuberculosis/.

[10] Page MJ, McKenzie JE, Bossuyt PM (2021). The PRISMA 2020 statement: An updated guideline for reporting systematic reviews. BMJ.

[11] (2024). Rayyan: AI-Powered Systematic Review Management Platform. https://www.rayyan.ai/.

[12] (2024). JBI Critical Appraisal Tools | JBI [Internet]. https://jbi.global/critical-appraisal-tools.

[13] (2024). SIGN: Checklists. https://www.sign.ac.uk/using-our-guidelines/methodology/checklists/.

[14] Kehbila J, Ekabe CJ, Aminde LN, Noubiap JJ, Fon PN, Monekosso GL (2016). Prevalence and correlates of depressive symptoms in adult patients with pulmonary tuberculosis in the Southwest Region of Cameroon. Infect Dis Poverty.

[15] Osman M, Welte A, Dunbar R, Brown R, Hoddinott G, Hesseling AC, Marx FM (2019). Morbidity and mortality up to 5 years post tuberculosis treatment in South Africa: A pilot study. Int J Infect Dis.

[16] Ayana TM, Roba KT, Mabalhin MO (2019). Prevalence of psychological distress and associated factors among adult tuberculosis patients attending public health institutions in Dire Dawa and Harar cities, Eastern Ethiopia. BMC Public Health.

[17] Hestad KA, Chinyama J, Anitha MJ, Ngoma MS, McCutchan JA, Franklin DR Jr, Heaton RK (2019). Cognitive impairment in Zambians with HIV infection and pulmonary tuberculosis. J Acquir Immune Defic Syndr.

[18] Ilesanmi OS, Adeniyi BO, Okunrinboye HI, Atoyebi AO, Erhabor GE (2020). The prevalence and factors associated with depression among patients with pulmonary tuberculosis at the Federal Medical Center, Owo, Nigeria. West Afr J Med.

[19] Philips L, Visser J, Nel D, Blaauw R (2017). The association between tuberculosis and the development of insulin resistance in adults with pulmonary tuberculosis in the Western sub-district of the Cape Metropole region, South Africa: A combined cross-sectional, cohort study. BMC Infect Dis.

[20] Nkereuwem E, Agbla S, Sallahdeen A (2023). Reduced lung function and health-related quality of life after treatment for pulmonary tuberculosis in Gambian children: A cross-sectional comparative study. Thorax.

[21] Binegdie AB, Parekh M, Tolessa TB, Ahmed FO, Sherman CB, Ethel JC, Schluger NW (2015). Sequelae of patients treated for pulmonary tuberculosis in chest clinic, Tikur Anbessa Specialized Hospital (TASH), Addis Ababa, Ethiopia. Ethiop Med J.

[22] Namusobya M, Bongomin F, Mukisa J (2022). Chronic pulmonary aspergillosis in patients with active pulmonary tuberculosis with persisting symptoms in Uganda. Mycoses.

[23] Kirenga BJ, Ssengooba W, Muwonge C (2015). Tuberculosis risk factors among tuberculosis patients in Kampala, Uganda: Implications for tuberculosis control. BMC Public Health.

[24] Aghanwa HS, Erhabor GE (1998). Demographic/socioeconomic factors in mental disorders associated with tuberculosis in southwest Nigeria. J Psychosom Res.

[25] Mkoko P, Naidoo S, Mbanga LC, Nomvete F, Muloiwa R, Dlamini S (2019). Chronic lung disease and a history of tuberculosis (post-tuberculosis lung disease): Clinical features and in-hospital outcomes in a resource-limited setting with a high HIV burden. S Afr Med J.

[26] Goussard P, Gie RP, Janson JT, le Roux P, Kling S, Andronikou S, Roussouw GJ (2015). Decompression of enlarged mediastinal lymph nodes due to mycobacterium tuberculosis causing severe airway obstruction in children. Ann Thorac Surg.

[27] Lasebikan VO, Ige OM (2015). Prevalence of psychosis in tuberculosis patients and their nontuberculosis family contacts in a multidrug treatment-resistant treatment center in Nigeria. Gen Hosp Psychiatry.

[28] Hassan WA, Darwish AM (2010). Impact of pulmonary tuberculosis on menstrual pattern and fertility. Clin Respir J.

[29] Hayes-Larson E, Hirsch-Moverman Y, Saito S, Frederix K, Pitt B, Maama-Maime L, Howard AA (2017). Depressive symptoms and hazardous/harmful alcohol use are prevalent and correlate with stigma among TB-HIV patients in Lesotho. Int J Tuberc Lung Dis.

[30] Theron G, Peter J, Zijenah L (2015). Psychological distress and its relationship with non-adherence to TB treatment: A multicentre study. BMC Infect Dis.

[31] Suthar AB, Lawn SD, del Amo J (2012). Antiretroviral therapy for prevention of tuberculosis in adults with HIV: A systematic review and meta-analysis. PLoS Med.

[32] Wondmeneh TG, Mekonnen AT (2023). The incidence rate of tuberculosis and its associated factors among HIV-positive persons in Sub-Saharan Africa: a systematic review and meta-analysis. BMC Infect Dis.

[33] Pachi A, Bratis D, Moussas G, Tselebis A (2013). Psychiatric morbidity and other factors affecting treatment adherence in pulmonary tuberculosis patients. Tuberc Res Treat.

[34] Castro-Silva KM, Carvalho AC, Cavalcanti MT (2019). Prevalence of depression among patients with presumptive pulmonary tuberculosis in Rio de Janeiro, Brazil. Braz J Psychiatry.

[35] Stoichita A, Dumitrescu A, Ciobanu A (2021). Depression and anxiety symptoms among people with rifampicin-resistant tuberculosis receiving in-patient care in the National Pulmonology Reference Institute in Romania. Monaldi Arch Chest Dis.

[36] Pasipanodya JG, McNabb SJ, Hilsenrath P (2010). Pulmonary impairment after tuberculosis and its contribution to TB burden. BMC Public Health.

[37] Stubbs B, Siddiqi K, Elsey H (2021). Tuberculosis and non-communicable disease multimorbidity: An analysis of the world health survey in 48 low- and middle-income countries. Int J Environ Res Public Health.

[38] Koyanagi A, Vancampfort D, Carvalho AF (2017). Depression comorbid with tuberculosis and its impact on health status: Cross-sectional analysis of community-based data from 48 low- and middle-income countries. BMC Med.

[39] Lönnroth K, Roglic G, Harries AD (2014). Improving tuberculosis prevention and care through addressing the global diabetes epidemic: from evidence to policy and practice. Lancet Diabetes Endocrinol.

[40] El Bcheraoui C, Mimche H, Miangotar Y (2020). Burden of disease in francophone Africa, 1990-2017: a systematic analysis for the Global Burden of Disease Study 2017. Lancet Glob Health.

